# Assisted ambulation to improve health outcomes for older medical inpatients (AMBULATE): study protocol for a randomized controlled trial

**DOI:** 10.1186/s13063-023-07501-y

**Published:** 2023-07-24

**Authors:** Joshua K. Johnson, Aaron C. Hamilton, Bo Hu, Quinn R. Pack, Peter K. Lindenauer, Robert J. Fox, Ardeshir Hashmi, Lee Anne Siegmund, Christian N. Burchill, Glen B. Taksler, Toyomi Goto, Mary Stilphen, Michael B. Rothberg

**Affiliations:** 1 Department of Physical Medicine and Rehabilitation, Neurological Institute, Cleveland Clinic, Cleveland, OH USA; 2 Center for Value-Based Care Research, Cleveland Clinic, Cleveland, OH USA; 3 Department of Hospital Medicine, Cleveland Clinic, Cleveland, OH USA; 4 Department of Quantitative Health Sciences, Cleveland Clinic, Cleveland, OH USA; 5 Department of Healthcare Delivery and Population Science, University of Massachusetts Medical School-Baystate, Springfield, USA; 6 Mellen Center for Treatment and Research in Multiple Sclerosis, Neurological Institute, Cleveland Clinic, Cleveland, OH USA; 7 Center for Geriatric Medicine, Cleveland Clinic, Cleveland, OH USA; 8 Office of Nursing Research and Innovation, and Consultant Staff, Lerner Research Institute, Cleveland Clinic, Cleveland, OH USA; 9 Penn Medicine Lancaster General Hospital, Lancaster, PA USA; 10 Population Health Research Institute, Case Western Reserve University at MetroHealth System, Cleveland, OH USA; 11 Rehabilitation and Sports Therapy, Neurological Institute, Cleveland Clinic, Cleveland, OH USA

**Keywords:** Ambulation, Activity, Mobility, Older adults, Geriatrics, Hospital, Inpatient

## Abstract

**Background:**

Hospitalized older adults spend as much as 95% of their time in bed, which can result in adverse events and delay recovery while increasing costs. Observational studies have shown that general mobility interventions (e.g., ambulation) can mitigate adverse events and improve patients’ functional status. Mobility technicians (MTs) may address the need for patients to engage in mobility interventions without overburdening nurses. There is no data, however, on the effect of MT-assisted ambulation on adverse events or functional status, or on the cost tradeoffs if a MT were employed. The AMBULATE study aims to determine whether MT-assisted ambulation improves mobility status and decreases adverse events for older medical inpatients. It will also include analyses to identify the patients that benefit most from MT-assisted mobility and assess the cost-effectiveness of employing a MT.

**Methods:**

The AMBULATE study is a multicenter, single-blind, parallel control design, individual-level randomized trial. It will include patients admitted to a medical service in five hospitals in two regions of the USA. Patients over age 65 with mild functional deficits will be randomized using a block randomization scheme. Those in the intervention group will ambulate with the MT up to three times daily, guided by the Johns Hopkins Mobility Goal Calculator. The intervention will conclude at hospital discharge, or after 10 days if the hospitalization is prolonged. The primary outcome is the Short Physical Performance Battery score at discharge. Secondary outcomes are discharge disposition, length of stay, hospital-acquired complications (falls, venous thromboembolism, pressure ulcers, and hospital-acquired pneumonia), and post-hospital functional status.

**Discussion:**

While functional decline in the hospital is multifactorial, ambulation is a modifiable factor for many patients. The AMBULATE study will be the largest randomized controlled trial to test the clinical effects of dedicating a single care team member to facilitating mobility for older hospitalized patients. It will also provide a useful estimation of cost implications to help hospital administrators assess the feasibility and utility of employing MTs.

**Trial registration:**

Registered in the United States National Library of Medicine clinicaltrials.gov (# NCT05725928). February 13, 2023.

## Administrative information

Note: the numbers in curly brackets in this protocol refer to SPIRIT checklist item numbers. The order of the items has been modified to group similar items (see http://www.equator-network.org/reporting-guidelines/spirit-2013-statement-defining-standard-protocol-items-for-clinical-trials/).

**Table:** 

Title {1}	Assisted Ambulation to Improve Health Outcomes for Older Medical Inpatients (AMBULATE): Study Protocol for a Randomized Controlled Trial
Trial registration {2a and 2b}.	Registered in the United States National Library of Medicine clinicaltrials.gov (# NCT05725928).
Protocol version {3}	1.0
Funding {4}	This study is funded by the National Institute on Aging (1R01AG073278-01A1).
Author details {5a}	Joshua K. Johnson^1,2^, Aaron C. Hamilton^3^, Bo Hu^4^, Quinn R. Pack^5^, Peter K. Lindenauer^5^, Robert J. Fox^6^, Ardeshir Hashmi^7^, Lee Anne Siegmund^8^, Christian N. Burchill^9^, Glen B. Taksler^2,4,10^, Toyomi Goto^2^, Mary Stilphen^11^, Michael B. Rothberg^2^ 1. Department of Physical Medicine and Rehabilitation, Neurological Institute, Cleveland Clinic, Cleveland, OH 2. Center for Value-Based Care Research, Cleveland Clinic, Cleveland, OH 3. Department of Hospital Medicine, Cleveland Clinic, Cleveland, OH 4. Department of Quantitative Health Sciences, Cleveland Clinic, Cleveland, OH 5. Department of Healthcare Delivery and Population Science, University of Massachusetts Medical School-Baystate, Springfield 6. Mellen Center for Treatment and Research in Multiple Sclerosis, Neurological Institute, Cleveland Clinic, Cleveland, OH 7. Center for Geriatric Medicine, Cleveland Clinic, Cleveland, Ohio 8. Office of Nursing Research and Innovation, and Consultant Staff, Lerner Research Institute, Cleveland Clinic, Cleveland, OH 9. Penn Medicine Lancaster General Hospital, Lancaster, PA 10. Population Health Research Institute, Case Western Reserve University at MetroHealth System, Cleveland, OH 11. Rehabilitation and Sports Therapy, Neurological Institute, Cleveland Clinic, Cleveland, OH
Name and contact information for the trial sponsor {5b}	Trial sponsor: National Institute on Aging Contact Name: Barbara Radziszewska (Program Official) Address: PO Box 8057, Gaithersburg, MD 20898 Phone: 800-222-2225 Email: radziszb@mail.nih.gov
Role of sponsor {5c}	NIA will monitor progress of the study and will convene the Data Safety Monitoring Board (DSMB). The PI will provide NIA with interval reports of study progress with enrollment, dropouts and safety events. If there are unexpected delays, NIA may offer advice or support. NIA will convene the DSMB and ensure it has regular meetings as determined by their charter. Data will be provided to the DSMB by the study statistician. NIA may stop the study at any point if it determines there are serious safety concerns or for evidence of futility. The study statistician will prepare an analysis for futility once 40% of the patients have reached Time 2.

## Introduction

### Background and rationale {6a}

Annually, there are approximately 12.8 million adults over the age of 65 hospitalized in the USA [1]. Hospitalization contributes to functional decline of older patients [2–6]. Inpatients spend as much as 95% of their time in bed, [7] and bed rest is associated with increased risk of complications such as falls, venous thromboembolism, skin breakdown, and hospital-acquired pneumonia [8, 9]. Such adverse events delay eventual recovery and increase costs as patients spend time in both acute and post-acute (e.g., skilled nursing and inpatient rehabilitation facility) care.

Frequent in-hospital physical therapy may contribute to improved function and discharge home, [10–12] but physical therapists primarily treat patients with significant functional impairments. They should not be the first-line clinicians for providing general mobility interventions (i.e., any activity out of bed, including ambulation) [13, 14]. Evidence is growing that general mobility interventions can be facilitated by non-therapists and may mitigate adverse events and improve patients’ functional status [9, 15–26]. The studies for this evidence base, however, are mostly observational and the effects are mixed. One randomized controlled trial has shown promise, but the effect on patients’ outcomes was assessed after hospitalization [27]. In each, interventions were facilitated by a bedside nurse or a designated mobility technician. Nurses, however, have multiple competing demands that often take precedence, [28] a problem exacerbated by the COVID-19 pandemic and resulting nationwide nursing shortage [29–31]. Additionally, fall prevention strategies—which are a necessary priority for nurses—often limit mobility out of bed [32]. Primarily for these reasons, ambulation is missed approximately 75% of the time even when it is ordered by a physician [7, 33–35].

A mobility technician (MT), a clinical team member with the sole responsibility of helping patients to regularly complete general mobility interventions, may be a viable solution to these problems. However, this is not a typical role in hospitals. A lack of empirical evidence that a MT can improve outcomes makes it challenging for hospital administrators to justify the creation of MT positions since doing so would increase costs for the hospital. There is a need for non-observational studies that examine the effect of a MT on both in-hospital and post-hospital outcomes.

## Objectives {7}

AMBULATE has three primary aims: first, to determine whether an ambulation program delivered by a MT improves patients’ mobility status at hospital discharge. As related objectives, we will examine if the intervention decreases hospital-related complications, including falls, venous thromboembolism, pressure ulcers, and hospital-acquired pneumonia; if it impacts discharge disposition (i.e., home vs. post-acute care facility); and if mobility improvement is sustained after hospital discharge. Second, we will use predictive modeling to identify which patients are more likely to benefit from this intervention. Third, we will assess the impact of the intervention on overall costs associated with the episode of care, including inpatient costs and medical costs following discharge.

## Trial design {8}

The AMBULATE study is a multicenter, single-blind, parallel control design, individual-level randomized trial. It builds from our pilot trials, which demonstrated that patients with a MT ambulated more than those receiving usual care, with a small increase in mobility status, reduction in length of stay, and an increased proportion discharging home [36, 37].

## Methods: participants, interventions, and outcomes

### Study setting {9}

The study will be conducted in five hospitals—four in the Cleveland Clinic Health System (Northeast Ohio, USA) and Baystate Medical Center (Springfield, MA, USA). Each hospital will employ 2 MTs to ensure that one is always available to provide the intervention.

### Eligibility criteria {10}

Medical (i.e., non-surgical) patients in the first 48 h of their admission will be identified by the MT, aided by an automatically generated daily report and collaboration with bedside nurses on the unit. This method increases the pragmatism of the study. Participants must meet all of these inclusion criteria upon review of the medical chart in the electronic health record:

1. ≥65 years of age.
2. Inpatient admission status to a medical service.
3. Complete history and physical examination on file in the electronic health record.
4. Moderate activity impairment based upon a raw score of 16–20 on the Activity Measure for Post-Acute Care (AM-PAC) 6-Clicks basic mobility short form. The 6-Clicks tool is a valid and reliable measure of function for patients in the acute hospital [38, 39]. Scores in this range are consistent with a patient who requires only a little physical assistance to complete basic mobility tasks like getting out of bed and walking, suggesting they are safe to get out of bed with a MT, but at risk of functional decline without assisted ambulation.
5. Insurance with traditional Medicare or Medicare Advantage. This will be required to determine post-hospitalization costs.

Exclusion criteria include:

1. Significant language barrier that requires a translator, except at Baystate where Spanish speakers may enroll since Spanish language translators are readily available.
2. Discharge planned within 24 h of screening.
3. Surgical procedure planned.
4. Diagnosed with unstable angina or other medical conditions precluding participation in exercise/ambulation.
5. Permanent residence in a skilled nursing facility.
6. Comfort care measures only.
7. Active infection with SARS-CoV-2 or other pathogen requiring contact or droplet precautions.
8. An order for bed rest that is clinically warranted due to safety concerns identified by a treating physician. Whether a bed rest order meets this criterion will be clarified by the MT with the nurse.
9. Not previously enrolled in the study.

### Who will take informed consent? {26a}

The MT will complete the informed consent process with any patient meeting inclusion/exclusion criteria. A patient who is unable to give consent can still be included if he or she otherwise meets inclusion/exclusion criteria, and a legally authorized representative gives consent. Our study has no inclusion or exclusion criteria related to race, ethnicity, sex, or gender.

### Additional consent provisions for collection and use of participant data and biological specimens {26b}

N/A. We will not collect biological specimens in this study. The potential for secondary use of data collected in this study is explained in the initial consent.

### Interventions

#### Explanation for the choice of comparators {6b}

Nurses are generally expected to perform a daily assessment of functional mobility and engage in goal setting and safe mobilization using appropriate resources, including nurse or family resources. In reality, practices vary substantially across and within nursing units on any given day due to competing needs. The randomized controlled trial design should make it more likely that intervention and control patients participate similarly in standard-of-care mobility with nurses, therapists, or family. In addition, we anticipate that on any given hospital unit there will likely be only 1 or 2 intervention patients at a time, thus decreasing the likelihood that nursing resources would be increased to assist control patients with ambulation as an unintended consequence of our study.

#### Intervention description {11a}

**Fig. 1:**
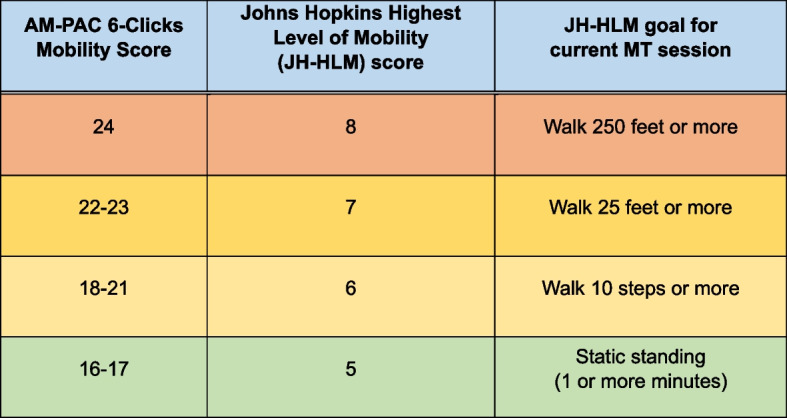
The Johns Hopkins Mobility Goal Calculator to guide the standardized MT protocol (figure adapted from Klein et al. [21], with permission)

Patients randomized to the intervention group will have up to 4 visits daily from the dedicated MT to ambulate according to a standardized mobility protocol with a goal of 3 walks per day. The protocol will be initiated within 24 h of the baseline assessment. It will employ the Johns Hopkins Mobility Goal Calculator (JH-MGC, Fig. 1), [21] which prescribes a mobility goal based on the Johns Hopkins Highest Level of Mobility (JH-HLM) scale considering the most recent 6-Clicks score. The MT will complete the 6-Clicks assessment within each completed visit (i.e., one in which the patient gets out of bed). Nurses have demonstrated successful use of the JH-HLM and JH-MGC as guides for goal-setting and mobility achievement in this population [21, 26]. Per the protocol, patients with a 6-Clicks score of 16–17 will aim to achieve at least standing near the edge of the bed for ≥1 min with assistance from the MT. Patients with a score of 18–20 will have a goal of walking ≥10 steps with the MT. The MT will adjust each session’s goal consistent with the JH-MGC, accounting for the most recently recorded 6-Clicks score from the MT. The goals represent minimum targets—many patients will accomplish more. If a patient demonstrates appropriate tolerance and safety, MTs will encourage walking greater distances.

MTs will continue to visit patients even if their 6-Clicks score increases to >20. However, the intervention will be paused if the patient scores <16, since these patients will require more skilled care than could be safely provided by the MT. In this case, MTs will continue to visit the patient daily and resume the intervention when the 6-Clicks score (recorded by a nurse or physical therapist) is ≥16. The intervention will terminate after the completion of the 10th day from the baseline assessment or the day of hospital discharge, whichever comes first and regardless of how many days the patient received the intervention. Prior to discharge, the MT will provide those in the intervention group with a standardized written guide for continuing to walk at home.

Compliance with the intervention will be assessed immediately following each patient encounter by recording the following: (1) service time and duration (minutes); (2) 6-Clicks score; (3) target level of activity from the JH-HLM; (4) activity level achieved from the JH-HLM; (5) physical assistance provided for ambulation (Yes/No); (6) assistive device used for ambulation (none, walker, cane, IV pole, supplemental oxygen, other); (7) if service failed, reason why (patient unavailable, patient refused, MT ran out of time, other); and (8) any falls and whether an injury occurred.

#### Criteria for discontinuing or modifying allocated interventions {11b}

We will temporarily discontinue the intervention if a patient has a clinical deterioration of 6-Clicks score below 16, and resume it once they return to 16 or higher. Similarly, if a patient has an order for bed rest, we will temporarily discontinue the intervention until the bed rest order is removed.

Subjects may withdraw voluntarily from participation in the study at any time and for any reason. Participants will continue to be followed, with their permission, even if the study intervention is discontinued. In that case, all follow-up evaluations will take place on the normal schedule.

#### Strategies to improve adherence to interventions {11c}

To complete up to three walks daily, the MT will check with each patient up to four times each day. In addition, each MT will complete training in motivational interviewing, [40] a skill that will be used to encourage ambulation when patients may be resistant.

To ensure fidelity across sites, we will follow best practices as recommended by the NIH Behavior Change Consortium [41]. We will ensure that the treatment “dose” is consistent by standardizing the number of attempts and using the JHH-MGC tool to set target distances via the written protocol. MTs will record each contact and its outcome as described above, as well as any protocol deviations. To minimize “drift” in MT skills, Dr. Johnson at CC and Dr. Pack at Baystate will conduct periodic observation. We will also arrange quarterly conference calls to share best practices, problem solve, and reinforce skills.

### Relevant concomitant care permitted or prohibited during the trial {11d}

No concomitant care will be prohibited during the trial. Patients may complete mobility activities with a nurse, family member, or, if consulted, an occupational or physical therapist.,

### Provisions for post-trial care {30}

There are no provisions for post-trial or ancillary care. There will not be any compensation for patients who may be injured during the trial.

### Outcomes {12}

The primary outcome will be change in mobility status at the conclusion of the intervention (hospital discharge or the 10th day of the intervention, whichever comes first) compared to the baseline assessment. Mobility status will be assessed at both time points using the Short Physical Performance Battery (SPPB), [42–44] a mobility performance assessment that can be administered in <10 min. It tests gait speed, repeated chair stands, and tandem balance, providing a gross assessment of functional balance, speed, and lower extremity power. It is safe and reliable for use with hospitalized older adults [45] and appropriately sensitive to mobility differences in that population [45, 46]. Further, the SPPB has demonstrated the ability to predict adverse health outcomes including in-hospital falls, [47] post-acute facility placement, [42] subsequent disability, [48] multi-morbidity, [42, 43, 49] and all-cause mortality [42, 50].

We will collect secondary outcomes at the time of hospital discharge including discharge disposition, length of stay, and the presence of hospital-acquired complications (falls, venous thromboembolism, pressure ulcers, and hospital-acquired pneumonia). Additional secondary outcomes will be assessed after hospital discharge and compared to baseline measurement of the same assessments: FRAIL scale score, [51, 52] the patient’s self-rated mobility status according to the AM-PAC mobility outpatient short form (compared to baseline AM-PAC 6-Clicks mobility scores using the AM-PAC T-scale), [53, 54] and the patient’s self-rated participation in activities of daily living using both the Index in Activities of Daily Living (Katz ADL) [55] and the Lawton Instrumental Activities of Daily Living Scale [56]. At this post-hospital time point, we will also assess 30-day hospital readmission and scores on the Patient Reported Outcomes Measurement Information System (PROMIS) physical function subscale [57, 58].

As both a secondary outcome and a method for characterizing the in-hospital mobility of both groups, we will use a medical grade accelerometer to measure steps taken, energy expenditure, activity bouts, sedentary bouts, body position, sleep latency, total sleep time, wake after sleep onset, and sleep efficiency. A random subset of 10–20% of patients discharged to home will continue to wear the device for an additional 10 days so that we can assess changes in activity immediately after discharge.

### Participant timeline {13}

**Fig. 2:**
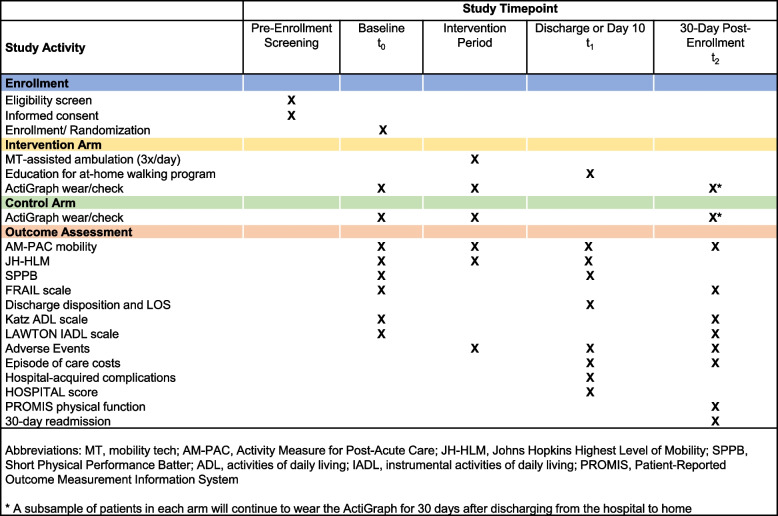
Participant timeline

The baseline assessment and randomization (T0) will occur for each patient within 48 h of hospital admission. They will be enrolled in the study until hospital discharge or after 10 days (T1), whichever occurs first. A post-discharge follow-up call from a study coordinator will be completed 30 days after enrollment (T2). This ensures that the time between T0 and T2 will be 30 days for every patient. The participant timeline, including assessments, is shown in Fig. 2.

### Sample size {14}

A sample size of 3000 has >99% power to detect a difference in SPPB change as small as 0.54 points (the MCID) between the two groups at a two-sided alpha of 0.05, assuming a common SD of 2.7 for the SPPB change, 20% non-compliance, 10% dropouts, and an interim analysis. The estimates for MCID and SD are based on observation of older acute care medical patients [43, 44]. Using these same assumptions, the power is still 92% for detecting a smaller difference of 0.35. Therefore, we expect ample statistical power for analyzing the primary outcome. The main reason for selecting this sample size is to ensure that the analyses of important secondary outcomes are not underpowered. The most important of these is discharge disposition, because even a small absolute difference in the number of patients discharged to home, as opposed to a skilled nursing facility, is clinically important. We will be able to detect a 7% difference in discharge to home vs. SNF with 98% power.

For other binary secondary outcomes (a combination of venous thromboembolism, pneumonia and falls, 30-day mortality, or 30-day readmission), the study will have 85% power to detect a 33% reduction assuming an 8% rate in the control group. Because these outcomes are not contingent on patient cooperation, we anticipate no dropouts. For secondary continuous outcomes such as length of stay, assuming 20% non-compliance rate, the power to detect a 4-h difference (0.167 days) is 95%.

Regarding the cost analysis, there is no accepted way of doing a power calculation. However, we tried adding $300 (an approximation of the cost of the intervention) to the costs of each of 1500 patients drawn randomly from a sample of 3000 Cleveland Clinic medical inpatients over the age of 65 with an AM-PAC 6-Clicks score between 16 and 20. We then performed a *t*-test on the log-transformed costs, as described in the economic analysis section, and we were able to detect the increase with a p-value of 0.03, indicating that should the intervention increase costs by an amount equal to the cost of the intervention, we should be able to identify it. Similarly, if the intervention decreases total costs by $300 or more (not including the costs of the intervention), we should have sufficient power to detect it.

### Recruitment {15}

Recruitment will be conducted by the MTs at the various study sites. At 7 am each morning, we will provide the MTs with a list of potentially eligible patients based on a computer query of current patients. The MTs will then review the EHR for additional eligibility criteria. Based on our pilot, approximately 1 in 6 patients will qualify for the study and most can be excluded through chart review. MTs will then approach potentially eligible patients to ask if they might be interested in participating in the study. Interested patients will be screened for eligibility and, if they meet criteria and consent, will be enrolled into the study. Based on both the Cleveland Clinic and Baystate pilots, we expect that at least 50% of the eligible patients approached will consent to participate. This rate does not reflect the acceptability of the intervention, which has been almost universal in our non-randomized pilots. Rather, it reveals patients’ hesitance to participate in clinical trials in general, including having to wear the accelerometer, share medical data with the study team, complete physical testing at baseline and at discharge, and report on mobility, frailty, and ADLs at 30 days, with only a 50% chance of receiving the active intervention. Our goal is to recruit 200 patients per year at each hospital. This represents approximately 1–2% of all medical admissions (depending on the hospital size). As a result, we anticipate there will be plenty of patients available for recruitment on any given day. MTs will be expected to recruit 4 patients per week or approximately 1 patient every other day. They should have sufficient time to provide ambulation assistance for 4 patients daily, but will generally have 2 or 3 patients to assist.

## Assignment of interventions: allocation

### Sequence generation {16a}

Randomization will be done using a computerized system. A block randomization scheme with a size of 4 will be used to ensure approximately equal numbers of patients per group. Allocation will be stratified within units within hospitals.

### Concealment mechanism {16b}

The block size will not be disclosed to study staff who are enrolling patients.

### Implementation {16c}

The allocation sequence is determined a priori by a biostatistician. The MT will be responsible for the randomization process and patient allocation.

## Assignment of interventions: blinding

### Who will be blinded {17a}

Blinding of patients and MTs to the intervention is not possible. However, outcomes at hospital discharge and after discharge will be collected by assessors blinded to allocation.

### Procedure for unblinding if needed {17b}

N/A. There is no indication of unblinding the blinded assessors. 

## Data collection and management

### Plans for assessment and collection of outcomes {18a}

#### Primary outcome: Short Physical Performance Battery

The initial SPPB will be administered by the MT enrolling the patient as part of the baseline assessment. The final SPPB will be administered by a physical therapist or exercise physiologist blinded to the patient’s treatment group allocation. All assessors will be trained by the primary study physical therapist (J.K.J.). Acceptable inter-rater reliability of the SPPB has been shown in prior studies, [59, 60] but we will assess inter-rater reliability for the first 10 patients enrolled with each mobility tech as a quality check; the primary study physical therapist (J.K.J.) will be the second assessor.

#### Secondary hospital clinical outcomes

Patients’ hospital discharge disposition, length of stay, and the presence of hospital-acquired complications (falls, venous thromboembolism, pressure ulcers, and hospital-acquired pneumonia) will be assessed retrospectively using medical record data, using a unique encounter number to identify study episodes. This number will also be matched to episode-level billing data to identify the cost of the hospital stay.

To characterize the mobility of both randomized groups, each patient will wear a medical grade accelerometer to directly record all movement throughout their hospital stay. The MT will place the accelerometer on the patient’s wrist like a watch (for better compliance and more accurate sleep measurement) with a sampling frequency of 90 Hz and an LFE filter. The device is highly water resistant and does not need to be removed for bathing. It will not display any information to the patient and the patient need not take any action. Raw acceleration data will be translated via algorithms into meaningful activities, including steps taken, energy expenditure, activity bouts, sedentary bouts, body position, sleep latency, total sleep time, wake after sleep onset, and sleep efficiency. Data will be downloaded from each device by the MT at least weekly and at discharge. Except for the random subset of 10–20% of patients who will continue to wear the device at home for an additional 10 days after discharging home, devices will be collected at discharge.

#### Secondary post-hospital outcomes

At 30 days post-discharge, a research assistant will contact participants to collect the additional secondary outcomes (FRAIL, Katz ADL, Lawton IADL, AM-PAC mobility, PROMIS physical function, and 30-day readmission). Contact will be initiated by subjects’ preferred means of communication, including telephone, email, patient portal, or text. Participants will be compensated for their time to respond. Based on preliminary efforts, we anticipate being able to contact 90% of the patients in 30 days. If we are unable to contact the patient, we will mail them the survey to complete on paper and return to our study team.

### Plans to promote participant retention and complete follow-up {18b}

#### Retention in the hospital

Each day, MTs will visit patients in the intervention arm until they have ambulated 3 times or the MT has made 4 attempts. They will consult with daily schedules to avoid visiting while patients are sleeping, eating, or having procedures. Daily step counts and other activities will be collected automatically from the accelerometer worn by the patient on the wrist. It is waterproof and can be worn continuously, so patients are not required to take any action to participate. In our pilot, we had very few patients withdraw during hospitalization. We do not anticipate this will be a problem. If it is, we will assess patient reasons for discontinuing and craft strategies to address those issues.

#### Retention after discharge

Participation in the trial includes consenting to being contacted at 30 days in order to complete surveys regarding mobility, ADLs, and frailty. Subjects will be asked at enrollment for their preferred means of contact, including telephone, email, patient portal, or text message. Patients who are discharged alive will be reminded at discharge that they will be contacted at 30 days and that they will be compensated $20 for their time. Members of the study team will make 3 attempts to contact each patient using all methods that the patient has supplied. In preparation for this study, we attempted to contact 20 patients who met our proposed inclusion criteria and were able to successfully engage 18/20 patients at 30 days. If we are not able to reach a patient by these means, we will examine the EHR and “Care Everywhere,” which includes records for all hospitals using EPIC, to see whether the patient is currently hospitalized. If all of these methods fail, we will send a registered letter. Once contact is made, the team member will remind the patient of the commitment that they made at enrollment to provide these data and of the importance of their data to the success of the study. They will thank them for their time and their contribution to the scientific endeavor. If a relative indicates that the patient is deceased or has been transferred to long-term care, we will note that. If possible, we will contact them in the long-term care facility. Patients who cannot be reached by any means or refuse to provide their data will be considered lost to follow-up. For patients who cannot be reached, we will attempt to identify their vital status and whether they are in a long-term care facility based on their Medicare claims data or the EHR.

### Data management {19}

Study data at all sites will be collected and managed using REDCap electronic data capture tools hosted at Cleveland Clinic [61, 62]. REDCap (Research Electronic Data Capture) is a secure, web-based software platform designed to support data capture for research studies, providing (1) an intuitive interface for validated data capture, (2) audit trails for tracking data manipulation and export procedures, (3) automated export procedures for seamless data downloads to common statistical packages, and (4) procedures for data integration and interoperability with external sources.

### Confidentiality {27}

Patient confidentiality will be maintained throughout the clinical study in a way that ensures the information can always be tracked back to the source data. For this purpose, a unique subject identification code (ID number and subject name code) will be used that allows the identification of all data (such as survey responses and scales) reported for each subject. Subject information collected in each phase of the proposed work will comply with the standards for the protection of privacy of individually identifiable health information as promulgated in the Health Insurance Portability and Accountability Act and as mandated in Title 45 CFR, Parts 160 and 164. All records will be kept confidential and no physician or patient name will be released by study staff at any time.

Returned paper surveys or telephone responses will be entered into REDCap for secure electronic storage, and paper copies of surveys will be locked in a cabinet in the offices of the Center for Value-Based Care Research. REDCap is protected behind a login and Secure Sockets Layer (SSL) encryption. Data collection is customized for each study or clinical trial based on a study-specific data dictionary defined by the research team. REDCap requires a password for entry. Patient-level data access will be available only to those members of the team directly involved in data collection or analysis. All electronic data systems are maintained behind the Cleveland Clinic firewall. Entry to the continually locked research area is restricted by a coded badge identification system.

### Plans for collection, laboratory evaluation, and storage of biological specimens for genetic or molecular analysis in this trial/future use {33}

N/A. We are not collecting biospecimens.

## Statistical methods

### Statistical methods for primary and secondary outcomes {20a}

#### Statistical analyses

The main analysis will follow the intention-to-treat principle. The primary outcome, change in the SPPB from baseline to discharge (or 10 days), will be compared between the groups using an independent samples *t*-test. The large sample size should ensure that any confounding variables are equally distributed between groups. However, if we identify that any baseline variables are unbalanced and also associated with the outcome, we will model SPPB using a linear mixed-effects model to control for potential confounding. The fixed effects will include group, measurement time point, their interaction, site, and any unbalanced baseline variables. Secondary binary outcomes (discharge disposition and hospital-acquired complications) will be assessed using the chi-square test. The secondary longitudinal outcome (mobility improvement 30 days after hospital discharge) will be modeled using linear mixed-effects models.

### Interim analyses {21b}

After 600 (40%) patients enrolled per group, we will conduct an interim analysis of the primary outcome to assess whether the study is working as expected. The O’Brien-Fleming boundaries at the interim analysis for efficacy and futility are 3.35 and −3.35, respectively. If the futility boundary is crossed, indicating that we have failed to improve patient mobility, then the trial will be stopped, because if we cannot improve mobility, there is no reason to think we could improve the other outcomes. On the other hand, if we cross the boundary for efficacy, we will still continue the trial in order to assess the important secondary outcomes which are the basis for our power calculation. We do not expect to assess outcomes such as discharge disposition until the end of the trial. The one exception is that halfway through the trial (at 18 months) we will compare the safety outcomes of falls and falls with injury. The DSMB will review these results to determine if there are significant risks to continuing the study.

### Methods for additional analyses (e.g., subgroup analyses) {20b}

We will conduct three sensitivity analyses to account for (1) missing outcomes data, (2) treatment heterogeneity by site, and (3) the dose-response curve in the intervention group.

To identify the patients most likely to benefit from ambulating with a MT, we will build models separately for each treatment arm to predict the outcome of at least 1-point increase in the SPPB (i.e., beyond the minimal clinically important difference). Using the predicted probabilities resulting from these models, we will develop a Classification and Regression Tree (CART) that should identify subsets of patients likely to benefit, not benefit, or be harmed by the treatment. For example, the analysis might show that female patients aged >75 years who have an initial 6-Clicks score between 16 and 18 have an estimated treatment benefit of being 22% more likely to achieve the 1-point increase in SPPB. Essentially the tree will form risk groups of patients with shared characteristics that should receive a similar care recommendation.

We will estimate the total cost of the episode of care including the index admission (from hospital cost accounting systems) and all non-medication costs for the 30 days following enrollment (from Medicare claims) for all patients. For the intervention group, we will add the personnel costs for the MTs (hourly wages plus benefits). Since cost data are skewed, our cost estimates will be transformed and then compared between groups using the *t*-test.

### Methods in analysis to handle protocol non-adherence and any statistical methods to handle missing data {20c}

Outcomes will be assumed missing at random in the main analysis. We will perform one sensitivity analysis in which we impute missing outcomes using a chained equation approach, and another using the marginal structure model (MSM), which can handle possible bias introduced by noncompliance in the intervention group and informative censoring/dropout simultaneously.

### Plans to give access to the full protocol, participant-level data, and statistical code {31c}

The investigators are committed to sharing the data from this study with the broader scientific community. As part of that effort, we will make our complete de-identified study data set, including study assignment, patient characteristics, and outcomes, available upon request from the Principal Investigator, 24 months after the conclusion of the randomized trial. Patient information will be de-identified in compliance with HIPAA regulations and requesters will be required to sign a data use agreement that prohibits them from attempting to reidentify participants. To the extent possible, case report forms for the study will be designed using Common Data Element conventions (https://www.nlm.nih.gov/cde/index.html) described in the subject areas of Demographics and Patient Contact Information, Medical History, and Study Details. All fields in the database will contain documentation to support the correct understanding of the data for internal and external data users.

## Oversight and monitoring

### Composition of the coordinating center and trial steering committee {5d}

The steering committee will meet monthly and be comprised of members of the study team, including experts in hospital medicine, physical therapy, nursing, organizational behavior, statistics, and health economics. There will be approximately 15 members. The committee will advise the PI on issues having to do with study design, decisions regarding implementation, data analysis, and interpretation. When the study is complete, the Steering Committee will oversee the publication of the manuscripts related to this work.

### Composition of the data monitoring committee, its role and reporting structure {21a}

The Data and Safety Monitoring Board (DSMB), composed of independent clinicians, will review the accumulating data with regard to recruitment, safety, and efficacy. The members of the DSMB will not be involved in the conduct of this study. The trial statisticians will summarize and report data to the DSMB semi-annually, and the DSMB will review the report and make recommendations to the investigators. Serious unexpected adverse events will be reported to the DSMB (and others) within 24 h of the study personnel learning of the events. NIA will recruit individuals to serve on the DSMB and schedule an initial meeting prior to starting the study. The purpose of the meeting will be to familiarize members with the protocol and to approve the DSMB charter, which will specify meeting frequency, data, and serious adverse event reporting requirements and stopping rules and guidelines.

### Adverse event reporting and harms {22}

We will monitor all adverse events and serious adverse events during the course of the study. Adverse events potentially related to the study will include any fall (defined as an event that results in a patient unintentionally coming to rest on the ground, floor, or other lower level), [47] which will be categorized as injurious or non-injurious; other musculoskeletal injuries; pulmonary embolism; and hospital-acquired infections. The principal investigator or his designee will assess each event for study-relatedness and severity. Serious adverse events will be reported to the Data Safety Monitoring Board (DSMB)

### Frequency and plans for auditing trial conduct {23}

The study coordinator at each site will audit the MT intervention logs and review step counts every month to ensure that participants are receiving the intervention, and they will provide feedback to the MT.

### Plans for communicating important protocol amendments to relevant parties (e.g., trial participants, ethical committees) {25}

Any protocol amendments deemed significant by the steering committee will be reviewed and approved by IRBs at both Cleveland Clinic and Baystate. These will also be shared with NIA.

## Dissemination plans {31a}

We plan to disseminate the results of the study widely and encourage their translation into practice in a number of ways, including through conference presentations (e.g., Society of Hospital Medicine and American Physical Therapy Association), peer-reviewed publications, and the creation of a Tool Kit. Embedded in the design of this project is the intent and plan to use the knowledge and products of the project to promote better care and minimize the harms of hospitalization for older adults. Results will be submitted to clinicaltrials.gov in a timely manner as specified by the NIH Policy on the Dissemination of NIH-Funded Clinical Trial Information.

### Local/regional dissemination

Members of the administration of our health systems are keenly interested in these results and would likely make these changes permanent if outcomes are favorable. Cleveland Clinic operates 15 hospitals in Northeast Ohio and Florida, while Baystate Health operates 4 hospitals in Western Massachusetts. Moreover, Cleveland Clinic is a national leader in healthcare innovation and holds annual meetings like the Innovation Summit. We will disseminate our findings at this meeting, and other similar meetings.

### National dissemination

To facilitate dissemination on a national scale, members of the study team will work with their respective professional societies, including the American Academy of Nursing, Gerontological Advanced Practice Nurses Association, Society for Hospital Medicine, American Physical Therapy Association, American Geriatrics Society, Association of Safe Patient Handling Professionals, and the American Society of Healthcare Risk Management (a subsidiary of the American Hospital Association) to promote the use of MTs through national guidelines and by influencing quality improvement organizations such as the Joint Commission and the National Quality Forum. Policy changes, including consensus guidelines and quality measures, are powerful catalysts for change at the health system level. By engaging appropriate thought leaders in national organizations, we will attempt to disseminate our findings through such policy changes. At the same time, we will work with various forms of media to disseminate our work. This will include the use of press releases to engage traditional media outlets and social media.

Finally, we will assemble a Tool Kit to facilitate the adoption of MTs by other health systems. The Tool Kit, which will be posted on the study website, will include materials necessary to start up a mobility tech program and carry out our intervention with high fidelity. It will include our MT training manual, instructions on how to license and use the 6-clicks instrument to determine patient eligibility, and will link to instructions on how to access and use the Johns Hopkins Mobility Goal Calculator. Study publications and links to other supporting articles will also be included.

## Discussion

In a 1947 issue of the British Medical Journal, Asher [63] advised against bed rest, writing: “Get people up and we may save our patients from an early grave.” Hirsch and colleagues [4] observed in 1990 that hospital-associated functional decline improved at a much slower rate than the acute illness. Such observations—now accumulated in multiple studies over more than 70 years—have led to labeling hospital-associated functional decline as an under-recognized epidemic [7] and identifying its role as part of the so-called post-hospital syndrome [64].

The primary objective of our study is to examine the extent to which ambulation interventions provided by an additional clinical team member—a MT—affects functional decline and other hospital-important outcomes (e.g., length of stay and discharge disposition) at costs that could be tenable for hospitals. Given current evidence, it is unclear if ambulation alone is enough to prevent the decline patients experience in the hospital. Further, we do not know how much ambulation may be required nor which patients benefit from ambulation, alone or in combination with other mobility interventions.

Appropriate exercise interventions in the hospital can, at the very least, attenuate functional decline. After cardiac surgery, ambulation facilitated by “ambulation orderlies” reduced functional decline and LOS [22, 37]. Kosse et al. [65] found in their systematic review that exercise interventions (provided by physical therapists or nurses) improved performance of physical function tests, contributed to fewer discharges to post-acute care facilities, and reduced hospital length of stay in a clinically heterogeneous sample of older adults. Similarly, in their single-center randomized controlled trial (*N*=370), Martinez-Velilla et al. [66] demonstrated that an individualized, multicomponent (resistance, balance, and basic mobility) exercise intervention in the hospital prevented functional decline and improved cognitive status.

Despite long-standing and ever-developing evidence that exercise could attenuate hospital-associated functional decline and lead to improved outcomes, physical activity interventions remain underutilized. This may be due, in part, to the relative complexity of these previously described interventions and the associated challenge of routinely implementing them in most hospitals. Additionally, usual care for hospitalized patients more often prioritizes fall prevention strategies that include bed rest—often with raised bedrails and activated bed alarms—over safe mobility practices [32]. With hospital quality metrics emphasizing fall prevention, [67, 68] and bedside nurses having multiple tasks that compete for their time and attention, [29] it is not surprising that patients only take, on average, 740 steps per day [33] and physician’s orders for ambulation are not completed up to 75% of the time [34, 69]. Indeed, nurses have reported concern about having limited time to provide mobility interventions, along with a lack of training and comfort with doing so [70].

Using MTs to assist with simple ambulation is consistent with the “culture of mobility” described as a goal for many hospitals, [71–73] in a way that does not further burden nurses. Early literature on the effects of in-hospital ambulation comes solely from observational or small prospective, randomized designs. One small nurse-led QI initiative demonstrated a 0.4-day decrease in LOS [70]. Another study noted that patients who walked more than 900 steps daily were less likely to experience functional decline (18% vs. 58%) [74]. A small randomized trial in the United States Veterans Health Administration (VHA) found that twice daily ambulation plus a behavioral strategy helped maintain post-hospitalization mobility [27]. An observational VHA study found that a daily walking program was associated with going home vs. a skilled nursing facility [18]. Our own prospective randomized controlled pilot trial in one hospital demonstrated that a MT could increase average daily steps from 668 to 994, with more intervention patients achieving 900 steps (28% vs. 19%). Additionally, intervention patients were more likely to go home [36]. No study, however, has used an experimental design to test the effect of MT-assisted ambulation on both in-hospital (including adverse complications) and post-hospital outcomes.

Our study will be the largest randomized controlled trial to test any exercise intervention for inpatients and tests a simple intervention (walking up to three times per day with a MT) that would be feasible for most hospitals to implement. There will be adequate statistical power to not only detect important differences in the primary outcome of mobility status, but also in less common hospital complications (falls, venous thromboembolism, pressure ulcers, and hospital-acquired pneumonia) that have been suspected to be due, at least in part, to unnecessary bed rest. We will also be able to develop a clinically useful classification model, allowing hospitals to recognize for whom ambulation with a MT may be most beneficial. This classification model will be strengthened by specific data on mobility dosing that we will collect from accelerometers. Lastly, our data will provide clear estimates of intervention costs relative to potential cost savings, information that will be important to help hospitals decide if implementation of a similar program is feasible for them.

Our study has limitations. First, the analyses of dose effect are observational and subject to confounding, as is the heterogeneity of treatment effect analysis to determine which patients benefit most. The cost analyses will suffer from some missing data. We may also have missing data for our primary outcome if patients go home without our knowing. Also, it is not clear whether the AM-PAC mobility measures can be reliably assessed by patient versus clinician raters at different time points without introducing measurement error [26, 75, 76].

Many potential limitations for our study are mitigated by the study’s design. For example, patients who are enrolled may be unable or unwilling to ambulate three times each day. If adherence is lower than expected in interim analyses, we will compare adherence rates across sites and attempt to identify and spread best practices. If that is not successful, we would still likely have sufficient power to detect a difference in our primary (>99% power with 70% adherence) and secondary outcomes (80% power with 70% adherence). Depending on the ease of enrollment, we may decide to enroll additional patients with the same resources. We anticipate heterogeneous patients, sites, and controls. Given our large sample, we anticipate that all of these important covariates will be balanced between the intervention and control arms. If we find that they are not, we will adjust our outcomes for important confounders. If we are unable to directly identify subgroups for whom the intervention is most beneficial, we will create a score, based on logistic regression, to identify patients who benefit if they score above a certain threshold. Alternatively, we will provide recommendations and tools to enable individual hospitals to incorporate our prediction model into the electronic health record and identify patients directly from the model.

While functional decline in the hospital is multifactorial, general out-of-bed mobility is a modifiable factor for many patients. This randomized controlled trial will test the effectiveness of MT-assisted ambulation on outcomes that matter to both patients and hospitals, including functional status, hospital discharge disposition, and the incidence of potentially avoidable hospital complications. It will also provide a useful estimation of cost implications to help hospital administrators assess the feasibility and utility of creating MT positions in their own hospitals.

## Trial status

Our study team is currently using protocol version 1 (dated April 25, 2023). Recruitment began on May 15, 2023, and is expected to conclude by May 31, 2026.

## Data Availability

The final trial data set will be retained by the study team, with the primary responsibility of its content and sharing lying with the Principal Investigator.

## Abbreviations

ADL: Activities of daily living
AM-PAC: Activity Measure for Post-Acute Care
CART: Classification and Regression Tree
DSMB: Data Safety Monitoring Board
IRB: Institutional Review Board
JH-HLM: Johns Hopkins Highest Level of Mobility
JH-MGC: Johns Hopkins Mobility Goal Calculator
MT: Mobility Technician
NIA: National Institute on Aging
PROMIS: Patient Reported Outcomes Measurement Information System
SPPB: Short Physical Performance Battery
